# Development of External Surfaces of Human Cerebellar Lobes in the Fetal Period

**DOI:** 10.1007/s12311-014-0566-3

**Published:** 2014-05-16

**Authors:** Marta Nowakowska-Kotas, Alicja Kędzia, Krzysztof Dudek

**Affiliations:** 1 Department of Neurology, Wrocław Medical University, ul. Borowska 213, 50-556 Wrocław, Poland; 2 Department of Normal Anatomy, Wrocław Medical University, Wrocław, Poland; 3 Department of Natural Sciences and Technology, Karkonosze College, Jelenia Góra, Poland

**Keywords:** Cerebellum, Cerebellar lobes, Cerebellar fissures, Fetal brain development

## Abstract

In the fetal period, development of cerebellar lobes may proceed dissimilarly due to possible differentiated origins of the cells and diversified times of their migration to certain cerebellum regions. This can cause various growth trajectories for the external surfaces of cerebellar lobes. The goal of the study was to describe the development of the external surface of cerebellum lobes and fissures delineating them in the fetal period. The material consisted of 101 fetuses (48 males and 53 females)—crown rump length 89–229 mm corresponding to 15–28 weeks of fetal life. The methods were based on anthropometric measurements and preparation techniques combined with elicited image computer analysis. At the largest values of the cerebellum posterior lobe surface, the most dynamic growth rate was observed in the case of the anterior lobe. Among the cerebellar lobes, proportional change was observed as well as a gradual increase in anterior lobe surface area and a simultaneous decrease in the surface area of the flocculonodular lobe part of the cerebellum total external surface. This paper presents the different growth trajectories of cerebellar lobes and demonstrates the importance of the primary fissure as a delineating mark for two regions with different dynamics of development.

## Introduction

The mechanisms of cerebellar development in the embryogenesis period are increasingly well-recognized, mainly at cellular and molecular levels. Its cells originate from two proliferative zones and their migration continues until the end of the first year of life [1]. Development of the cerebellum occurs with some delay in relation to the cerebrum. This delay in cerebellum linear size growth is observed until the fifth month of gestation, with a subsequent doubling of mass between the 19th and 35–37th weeks of fetal life [2–5]. Later, the cerebellum continues to develop dynamically and doubles its mass in relation to the cerebrum mass from 1:25 at the moment of birth up to 1:10–1:15 in adult individuals. The cerebellum development rate is determined by the external granular layer cell proliferation rate. This layer surface undergoes considerable augmentation due to the process of cortex enfoldment [6, 7] accompanied by the development of gyruses, the amount of which is estimated at 400–600 within the cerebellum external surface and in its fissures [8]. The first fissures appear on the external surface in the 12th week of fetal life. At first, they are most distinct within the cerebellar vermis. Intravital techniques (ultrasound examination and MRI) enable fissure visualisation with some delay (in the 27th–30th and 24th–32nd week, respectively) [9–11].

Two fissures are especially significant, because they delineate the individual lobes. The primary fissure separates the anterior from the posterior lobe and the posteriolateral fissure separates the posterior lobe from the flocculonodular lobe. On the basis of the literature discussing the origin of cerebellar cells, it has been suggested that the primary fissure could be the boundary separating the two regions’ cells originating from the mesencephalon and from metencephalon [12–15, review in 16]. Both the dynamic of development of the cerebellar lobes and the fissures in the fetal period seem to be areas of great interest.

Despite recent improvements in fetal MRI processing, the anatomical approach to the examination of cerebellum still offers a more adequate estimation of the external surface and fissures [9–11]. Here, we investigate, by means of anatomical and computer-enhanced methods, the external surface development of cerebellar lobes as well as the geometry of fissures delineating them in the fetal period. We hypothesized that cerebellar regions would show distinct growth curves and that the shape of the fissures would change.

## Material and Methods

The material consisted of 101 fetuses (48 male, 53 female), with the crown rump length (CRL) ranging from 89 to 229 mm, which corresponded to 15–28 weeks of gestation according to the Scammon and Calkins scales [17]. The number of fetuses in the fourth month amounted to six (four females, two males), in the fifth month to 43 (22 females and 21 males), in the sixth month to 29 (16 females and 13 males) and in the seventh month it amounted to 23 (10 females and 13 males). The sizes of the groups in particular weeks of fetal life are presented in Table 1. The selected fetuses, derived from the Normal Anatomy Department of Wrocław Medical University, did not reveal morphological manifestations of developmental anomalies. An examination of the karyotype of each fetus was not conducted.

**Table 1:** Fetus numbers in particular months and weeks of fetal life

Month	Week	Total	Female	Male
IV	15	3	2 (3.8 %)	1 (2.0 %)
	16	3	2 (3.8 %)	1 (2.0 %)
V	17	13	5 (9.6 %)	8 (16.3 %)
	18	11	8 (15.4 %)	3 (6.1 %)
	19	7	3 (5.8 %)	4 (8.2 %)
	20	12	6 (11.5 %)	6 (12.2 %)
VI	21	12	5 (9.6 %)	7 (14.3 %)
	22	7	5 (9.6 %)	2 (4.1 %)
	23	5	2 (3.8 %)	3 (6.1 %)
	24	5	4 (9.6 %)	1 (2.0 %)
VII	25	9	5 (9.6 %)	4 (8.2 %)
	26	9	3 (5.8 %)	6 (12.2 %)
	27	4	2 (3.8 %)	2 (4.1 %)
	28	1	0 (0.0 %)	1 (2.0 %)
	Total	101	52 (100 %)	49 (100 %)

Pearson Chi-square Test: *χ* ^2^ = 9.42, *df* = 13, *p* = 0.740

The following methods were used in the study: anthropological and dissection methods, digital image acquisition followed by measurements made by graphics programs and statistical methods. The superior and inferior surfaces of the cerebellum were exposed with the use of a stereomicroscope and microsurgical instruments. The anthropological method consisted of the assessment of fetal age by measuring somatic CRL. Photographic documentation showing superior and inferior surfaces of the cerebellum in horizontal positions was made with the use of a digital camera (Sony α 100—10 MP with 70–300 macro lens). Data were graphically processed (correction of image definition, contrast, frame) with the GIMP computer programme—version 2.6.9 (GNU Image Manipulation - http://www.gimp.org).

**Fig. 1:**
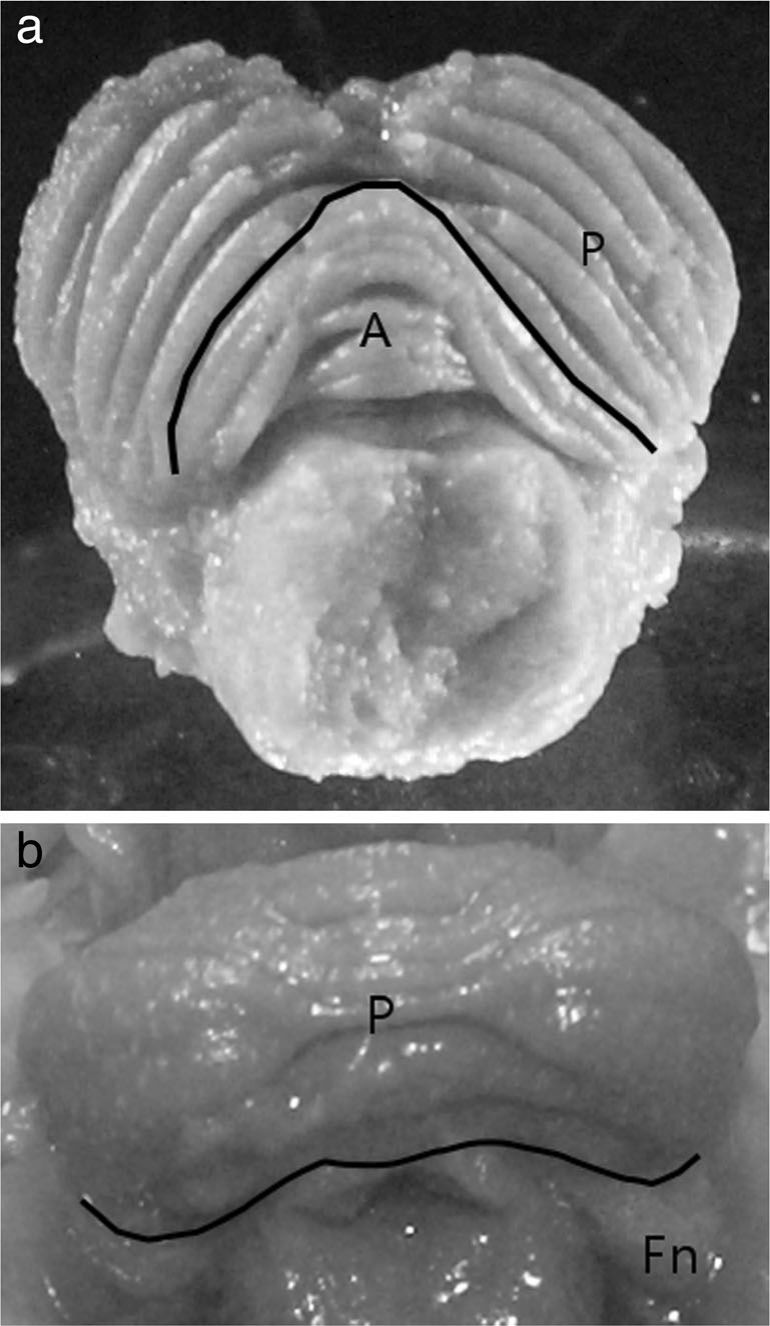
Schematic presentation of cerebellar lobe boundaries: **a** primary fissure separating the anterior lobe (*A*) from posterior lobe (*P*) (superior view), **b** posterolateralis fissure separating the posterior lobe (*P*) from flocculonodular lobe (*Fn*)

Linear measurements of surface areas and fissure enveloping curves (Fig. 1) were conducted using Scion Image for Windows (National Institute of Mental Health—NIMH, http://rsb.info.nih.gov/nih-image/download.html) and Universal Desktop Ruler—version 3.5.3364 (Avpsoft, http://avpsoft.com). Each image was scaled using the millimetre scale applied to every specimen. All the areas of lobes and surfaces were delineated visually according to the anatomical boundaries and were manually measured three times and in two planes (superior and inferior) by one researcher (M.N.K.). The average value was considered in data analyses. Intraobserver agreement was satisfactory, as the error estimated by the coefficient of variation was less than 2 % (Table 2).

**Table 2:** Coefficient of variation with intra individual variability measurement

Variable	Measurement (mean)			Mean ± SD	Coefficient of variation (%)
	1	2	3		
*P*_s_ (mm^2^)	100.9	99.3	99.0	99.7 ± 0.8	0.8
*P*_i_ (mm^2^)	87.2	89.0	87.5	87.9 ± 0.8	0.9
*P*_a_ (mm^2^)	30.4	30.3	30.7	30.5 ± 0.2	0.6
*P*_p_ (mm^2^)	115.2	115.0	115.7	115.3 ± 0.5	0.4
*P*_fn_ (mm^2^)	11.8	11.4	11.5	11.5 ± 0.2	1.7

*P*_*s*_ cerebellar superior surface area, *P*_*i*_ cerebellar inferior surface area, *P*_*a*_ anterior lobe surface area, *P*_*p*_ posterior lobe surface area, *P*_*fn*_ flocculonodular lobe surface area

**Fig. 2:**
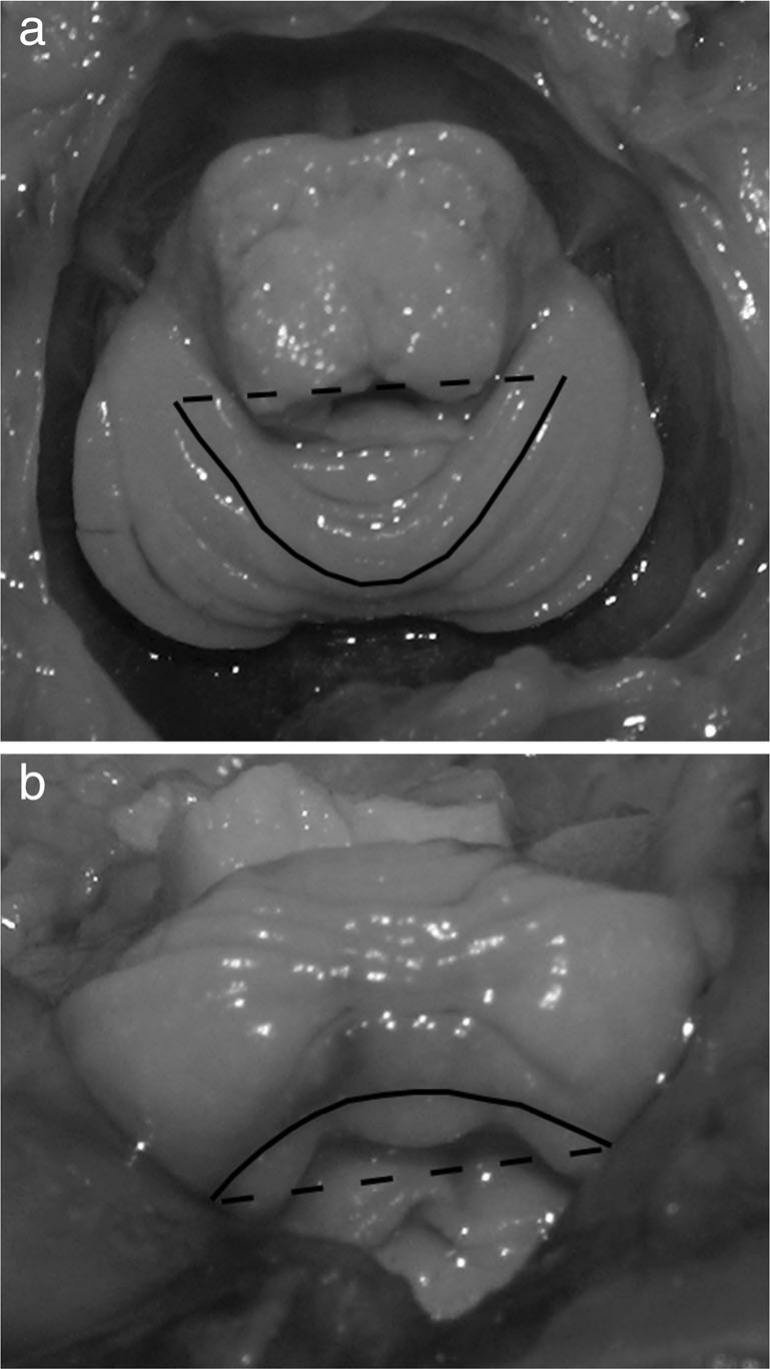
Schematic presentation of linear measurements of fissures: envelope of fissure (*L solid line*) and the shortest line joining the end points of the fissure (*x dotted line*) of **a** primary fissure (superior view) and **b** posterolateralis fissure (posterior view)

The analysis of fissure shape consisted of the measurement of the fissure envelope (*L*) and the shortest line (*x*) joining the end points of the fissure (Fig. 2). Based on the ratio of these two parameters (*L*/*x*), an analysis of the change in the curvature of the fissure could be made. Two fissures separating the particular lobes were included in the analysis: the primary fissure and the posterolateral fissure.

### Statistical Methods

The area measurements and linear measurements of the fissures of the cerebellar lobes were plotted against gestational age and the most appropriate model of growth (linear or exponential) was applied. The regression lines of the investigated features were plotted and the gradients of each line, representing a change either in area or in coefficient *L*/*x* per week, were calculated in order to compare growth rates. The Pearson coefficients in the case of the normal distribution features and the Spearman’s ratio were calculated to assess the correlations between areas and gestational age.

Ratios were calculated both between the areas of particular lobes, as well as between the area of the superior and inferior surfaces of the cerebellum. An analysis of variance (one-way ANOVA) was conducted. To establish the presence of sexual dimorphism, a comparison of the average values of the investigated features in sex subgroups was conducted either with the use of the *t* student test for independent variables for normal distribution features or with the use of the non-parametric *U* Mann-Whitney test in the other cases. The critical significance level was established as *p* = 0.05. The STATISTICA v.9 (StatSoft Inc., Tulsa, USA), Exel and MedCalc (MedCalc Software, Ostend, Belgium) statistical programs were used in the calculations. The Bioethical Committee of Wroclaw Medical University approved the study.

## Results

The cerebellar surface area of the cerebellum, calculated as the sum of the anterior, posterior and flocculonodular lobe surface areas, increased on average by 3.5-fold from the 15th to 28th week of gestation, and fitted an exponential model of growth according to the equation (Table 3):

**Table 3:** Growth of whole external surface of cerebellum calculated as sum of surfaces: anterior lobe (*P*_a_), posterior lobe (*P*_p_) and flocculonodular lobe (*P*_fn_) with reference to age

Age (months)	*P*_a_+*P*_p_+*P*_fn_ (mm^2^)		Percent
	Mean	SD	
4	75.7	9.0	100
5	112.8	31.0	149
6	175.2	38.7	231
7	267.2	63.1	353

$$ Pa+ Pp+ Pfn=49.2\cdot {e}^{0.4224{\cdot}_{age}} $$

**Fig. 3:**
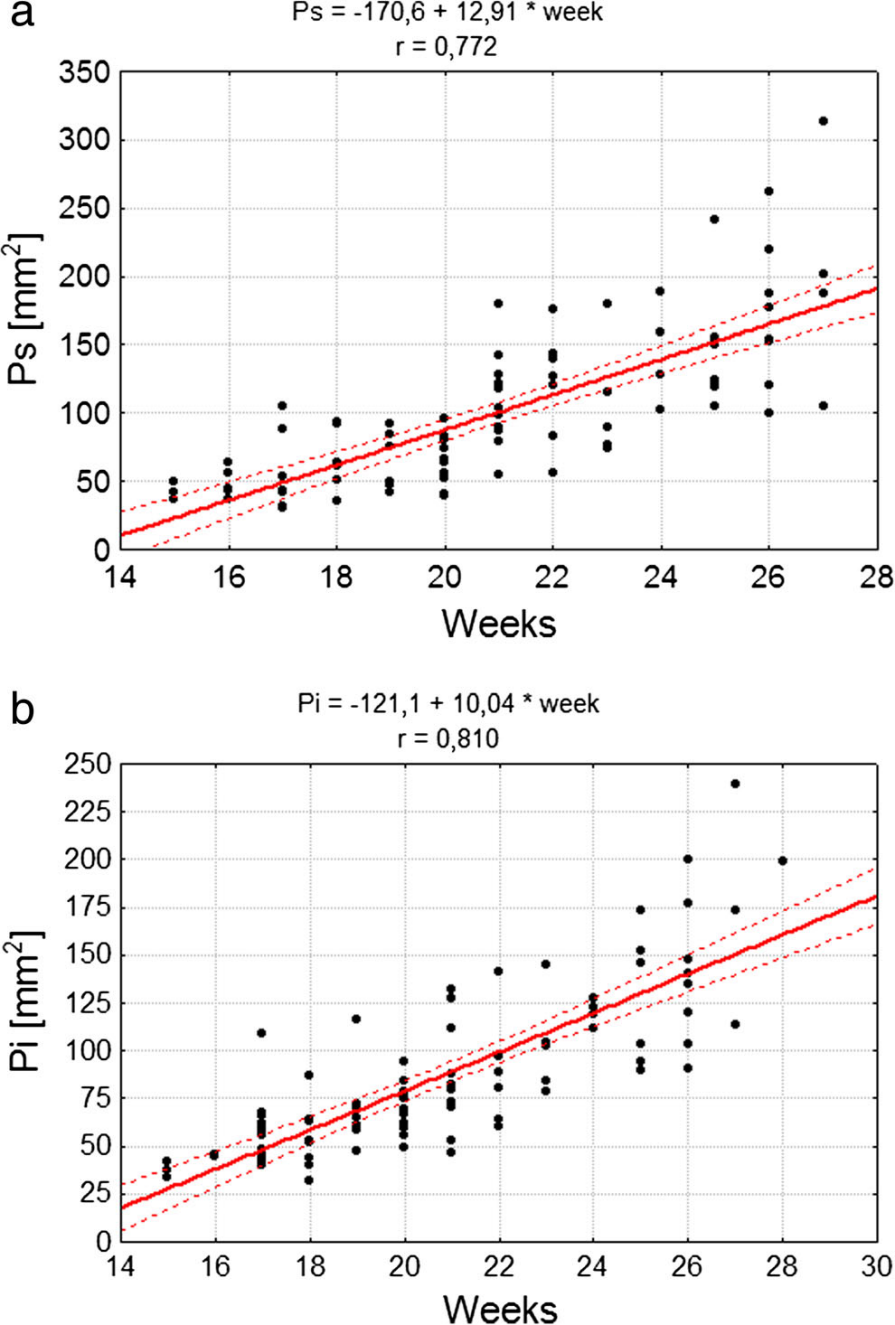
Correlation diagram of analysed features and week of fetal life, as well as parameters of the model of feature variability along with age for **a** *P*_s_ area of superior surface of cerebellum, **b** *P*_i_ area of inferior surface of cerebellum

The growth rate of the superior (*P*_s_) and inferior (*P*_i_) surfaces of the cerebellum best fitted the linear model of development (Fig. 3).

**Fig. 4:**
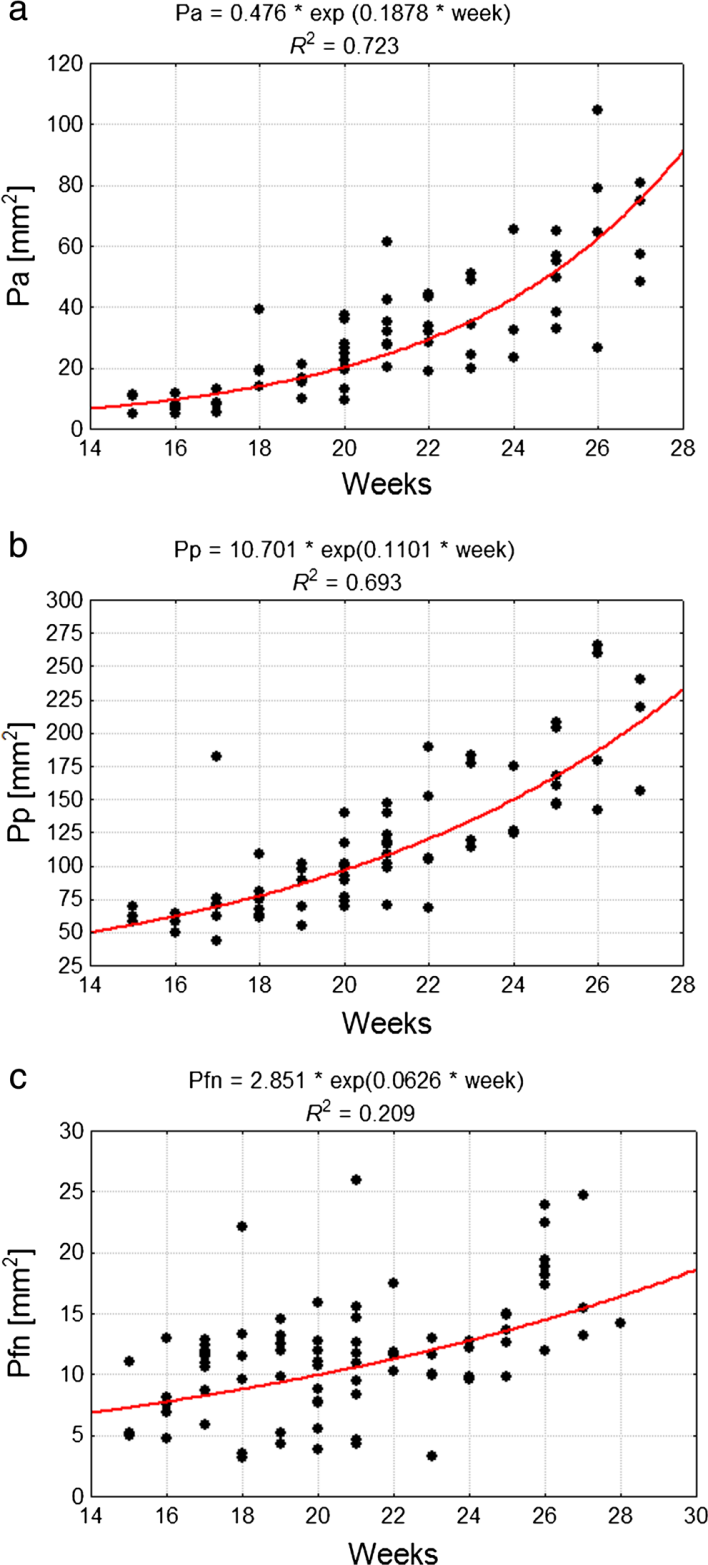
Correlation diagram of analysed features and week of fetal life, as well as parameters of the model of feature variability along with age for: **a** *P*_a_ anterior lobe surface area, **b** *P*_p_ posterior lobe surface area, **c** *P*_fn_ flocculonodular lobe surface area

Over the whole examined fetal period, the cerebellar posterior lobe (*P*_p_) had the largest values for surface area, followed by the anterior (*P*_a_) and then flocculonodular lobe (*P*_fn_). The average value for *P*_a_ increased from 7.62 mm^2^ in the 4th month up to 59.52 mm^2^ in the 7th month of gestation, for *P*_p_ and *P*_fn_ those values were 60.46 and 192.19 mm^2^, and 7.65 and 16.62 mm^2^, respectively. The growth rate of areas of all lobes steadily increased; therefore, subsequent analysis was based on an exponential model of growth (Fig. 4). The largest increases were noted in the case of *P*_p_ (Fig. 4b) and the smallest in the case of *P*_fn_ (Fig. 4c). However, *P*_a_ revealed the most dynamic growth rate among all the observed lobes between the fourth and seventh months of fetal life (exponential coefficient: 0.1878 versus 0.1101 for *P*_p_ and 0.0626 for *P*_fn_) and the ratio of *P*_s_ increased from 16 to 35.5 % over this period of development (Fig. 4a).

**Fig. 5:**
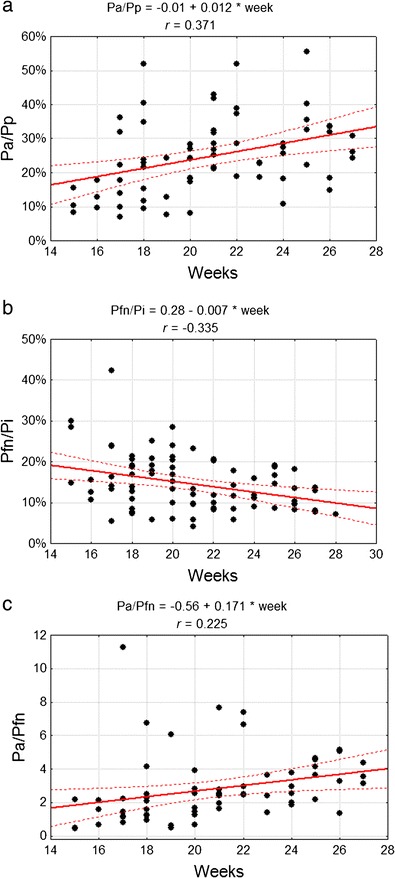
Correlation diagram of analysed features with the week of fetal life **a** dimension ratio of anterior (*P*_a_) and posterior cerebellar (*P*_p_) lobe areas **b** dimension ratio of flocculonodular lobe (*P*_fn_) and cerebellar inferior (*P*_i_) surface areas **c** dimension ratio of anterior lobe (*P*_a_) area and flocculonodular lobe (*P*_fn_) area

**Fig. 6:**
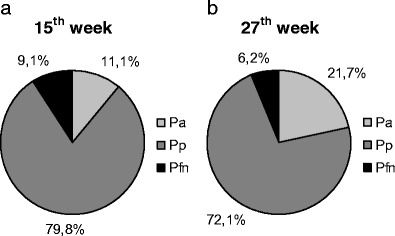
Proportions of the anterior (*P*_a_), posterior (*P*_p_) and flocculonodular lobe (*P*_fn_) at **a** 15th and **b** 27th weeks of gestation

**Fig. 7:**
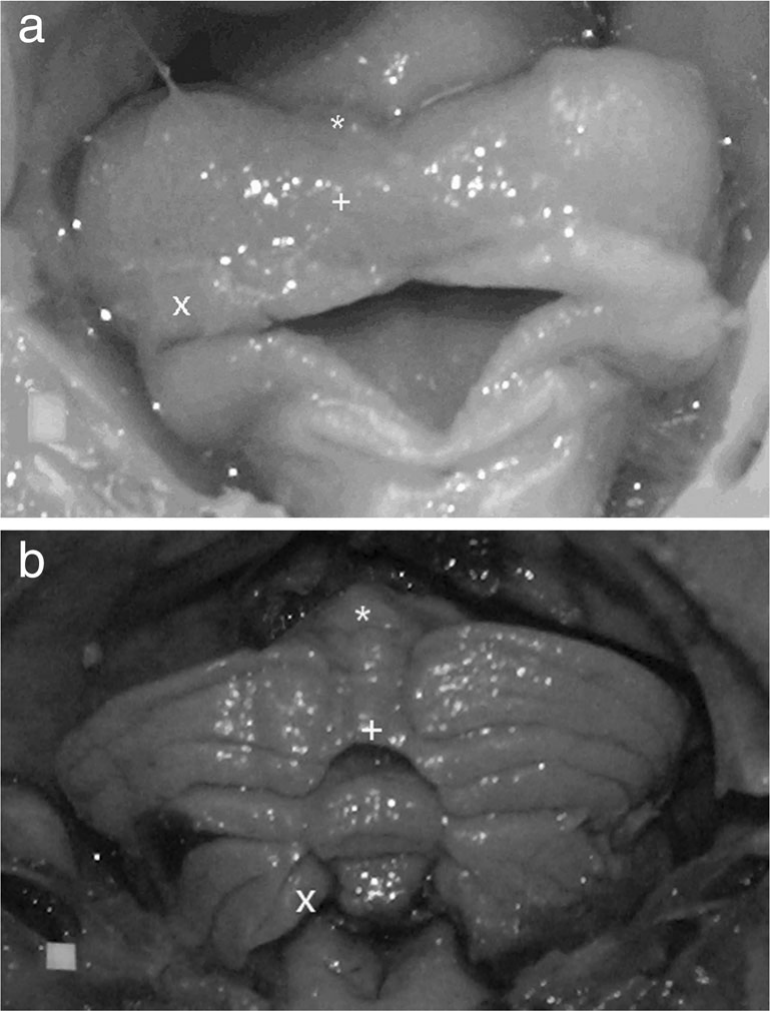
Shape of the human cerebellum at **a** 15th week and **b** 27th week of gestation (posterior view), with marked anterior lobe (*asterisk*), posterior lobe (*plus sign*) and flocculonodular lobe (*ex symbol*). Scale *white square* is 1 mm^2^

Dimension indices were calculated in order to discount any influence of conservation on the elicited results. These results revealed the interlobular area proportion change with reference to *P*_s_, *P*_i_ and the whole surface area (calculated as the sum of *P*_a_, *P*_p_, and *P*_fn_). The *P*_a_/*P*_p_ ratio increased significantly from 0.12 in the group of the youngest fetuses to 0.31 (*p* < 0.005) in the group of the oldest ones, where a changed was manifested in the relationships between the anterior and posterior lobe areas (Fig. 5a). At the same time, the *P*_a_/*P*_s_ ratio increased from 0.17 in the youngest group to 0.35 in the oldest one (*p* < 0.05). The *P*_fn_/*P*_i_ ratio decreased from 0.19 in the youngest group to an average of 0.12 (*p* < 0.05) in the oldest group, which represents a decrease of the flocculonodular lobe area in relation to the cerebellar inferior surface area (Fig. 5b). However, the *P*_a_/*P*_fn_ ratio and ratios between the *P*_p_, *P*_fn_ and *P*_i_ did not change (Fig. 5c). A comparison between the particular lobe areas and the whole surface area revealed the dominant contribution of *P*_p_ during the whole examined period, with its ratio constantly dropping (Table 4). Simultaneously, the role of *P*_a_ in the whole surface area increased and the ratio of *P*_fn_ decreased (Fig. 6), causing a change of the cerebellar shape (Fig. 7).

**Table 4:** Ratios of the lobes areas in whole surface of cerebellum calculated as sum of surfaces: anterior lobe (*P*_a_), posterior lobe (*P*_p_) and flocculonodular lobe (*P*_fn_) with reference to age

Week	*P*_a_/(*P*_a_+*P*_p_+*P*_fn_)	*P*_p_/(*P*_a_+*P*_p_+*P*_fn_)	*P*_fn_/(*P*_a_+*P*_p_+*P*_fn_)
15	0.111 ± 0.040	0.798 ± 0.016	0.091 ± 0.051
16	0.088 ± 0.016	0.800 ± 0.032	0.112 ± 0.044
17	0.096 ± 0.026	0.791 ± 0.064	0.113 ± 0.046
18	0.195 ± 0.044	0.734 ± 0.030	0.070 ± 0.050
19	0.147 ± 0.026	0.759 ± 0.056	0.094 ± 0.061
20	0.188 ± 0.077	0.731 ± 0.094	0.081 ± 0.030
21	0.214 ± 0.056	0.709 ± 0.054	0.077 ± 0.031
22	0.179 ± 0.029	0.746 ± 0.048	0.075 ± 0.024
23	0.193 ± 0.073	0.746 ± 0.065	0.052 ± 0.021
24	0.200 ± 0.057	0.742 ± 0.046	0.058 ± 0.018
25	0.223 ± 0.032	0.718 ± 0.023	0.058 ± 0.013
26	0.226 ± 0.094	0.707 ± 0.091	0.067 ± 0.015
27	0.217 ± 0.018	0.721 ± 0.032	0.062 ± 0.015

*P*_a_ and *P*_p_ strongly correlated with age (*r* amounted to 0.79 and 0.80, respectively), whereas *P*_fn_ correlated with age to a lesser degree (*r* = 0.55); however, the correlation was still statistically significant.

No sexual dimorphism was noted for any of the examined features.

**Fig. 8:**
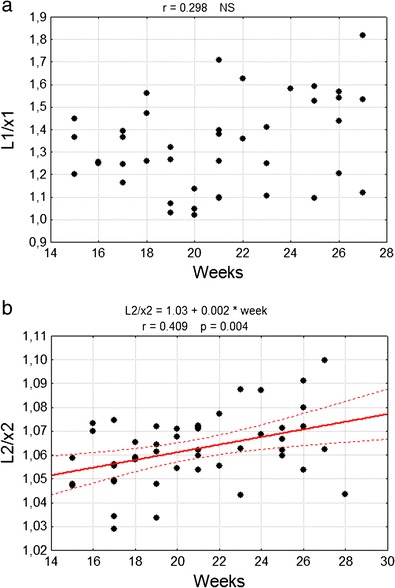
Correlation diagram of analysed coefficient *L*/*x* with the week of fetal life of **a** primary fissure (*L1/x1*), **b** posterolateral fissure (*L2/x2*). *NS* non statistically important

The curvature of the primary fissure estimated by the coefficient *L*/*x* did not change during the investigated fetal period (Fig. 8a). The same coefficient, in the case of the posterolateral fissure, increased in a statistically important manner from 1.05 to 1.07 in the examined material (*p* < 0.05) (Fig. 8b).

## Discussion

The 3.5-fold increase in the whole cerebellar surface area observed from gestation weeks 15 to 28 is smaller than the 30-fold increase in the external cerebellar surface area reported by Lemire et al. [18], who also took into account the surface of the cortex inside the fissures. The exponential model of all lobular surface area growth results from the simultaneous growth of the examined structures in terms of width and length. The relative proportions of the surface area change over time and indicate a change of cerebellar shape. Models of cerebellar area development in ultrasonographic studies differ widely from linear for the cerebellar transverse surface [4] to parabolic for the area of the sagittal section of hemispheres [19].

The biggest increase which is seen in the surface area characteristic for the posterior lobe and also changes of area indices indicating the most dynamic growth rate of the anterior lobe do not constitute any contradiction. Despite the dynamic development of the anterior lobe in the examined period, this was found to be smaller than the posterior lobe during the whole examined period. This is consistent with observations made by Adamsbaum et al. [20], who estimated the anterior and posterior lobe ratio to be 1:2, based on sagittal sections of fetal cerebellum in an MRI study.

An increase in the *P*_a_ proportion in terms of the total area of the cerebellum superior surface, and a simultaneous decrease in the *P*_fn_ proportion in the inferior surface area, as well as a change of the proportions between these two lobes point to the dynamic development of the cerebellum superior surface in the examined period of fetal life, with the most dynamic development (expressed by the highest coefficient of the growth trajectory) being in the cerebellum anterior lobe. Subsequently, the *P*_p_ was observed to develop more dynamically than the *P*_fn_. The flocculonodular lobe’s share of the total *P*_i_ was found to decrease with time and this may result from many factors. First of all, it is a structure which, in the course of its development, is covered by the cerebellum posterior lobe, so its development is more easily followed on cerebellum axial or transverse sections than on the cerebellum external surface. The change of the shape of the inferior part of the vermis in comparison with adjacent parts of hemispheres, along with the increase in its concavity, has been intravitally described with the use of MRI techniques [21]. This also remains consistent with our observation that the posterolateral fissure increases in curvature during the fetal period. Secondly, this structure is philogenetically old, so the lobe may be readily distinguished on the cerebellum external surface, and it may soon reach its final size. In addition, cerebellum inferior regions receive granular cells from the internal granular layer later than superior regions [22]; thus, we cannot exclude the presumption that, in the later period (not observed in this study), flocculonodular lobe growth could be more dynamic.

This paper demonstrates the different developmental trajectories of superior parts of the cerebellum and the importance of the primary fissure as a delineating mark. The results remain consistent with observations made on the basis of intravitral examinations [15, 23]. The paper highlights the different biology of the cerebellum superior region. Based on studies performed on animals, it can be assumed that Purkinje cells, by changing their orientation in early gestation and spreading primarily in the anteroposterior plane [24], could differentiate the growth rates of particular lobes. Granule cells could also influence the shape of the cerebellum, while they preferentially migrate first to anterior folia, and it is hypothesized that their transverse migration could regulate the formation of folia [25]. In the context of the most recent molecular examinations, this observation can be associated with the effect of the further dislocation of the coarctation organizer in a caudal direction in the late fetal period [16]. On the other hand, this may be the effect of cell origins being from the two different regions of the cerebrum (mesencephalon and metencephalon), as some papers based both on chick/quail chimera analysis as well as on the presumption that isthmus location is stable have claimed [12, 13 review in 16]. Based on the collected data, we cannot differentiate between those two theories. In both cases, the primary fissure separating cerebellar anterior and posterior lobes plays the role of a structure separating the regions of two different developmental dynamics, although it itself does not change shape in the examined period of fetal life.

The exploratory technique applied in this study eliminates the image and actual status incompatibility connected with non-invasive visualising techniques. In comparison to ultrasonographical techniques, it offers a more appropriate estimation of hemisphere external surface and earlier detection of fissures. In comparison with MRI imaging, it has proved to be more exact in terms of the differentiation of particular fissures on the external surface. In all the examined fetuses, the primary and posterolateral fissures were noted by means of the anatomical technique. This is more sensitive than MRI techniques, where differentiation of the first fissures in the 24th week of gestation and the impossibility in all cases to distinguish the flocculonodular lobe, even up to the end of gestation, are reported [10, 11]. More recent papers using advanced MRI techniques have concentrated on volumetric measurements and have not raised the issue of fissures and areas of external surfaces [21, 23].

However, the anatomical technique is connected with a number of difficulties. One of them is the susceptibility of the specimens to the soft tissue conservation effect which is minimalized by the introduction of dimensional indices. Another difficulty is the evaluation of external surface areas without an analysis of axial sections or histological examinations, as they could complete our understanding of the mechanisms of particular lobe development. It seems that the inclusion of microscopic techniques might enable the correlation of the external surface development process with external granular layer cell multiplication and migration. In turn, axial sections would enable simultaneous assessment of the hemispheres and cerebellar vermis cortex area located in fissures.

Our acquired additional understanding of the diversification of cerebellar lobule growth rates may improve estimations of gestational age using other techniques which enable in utero examinations. This may also have a considerable clinical value, as it may explain some of the cerebellar malformations and growth retardations which occur in the fetal and perinatal periods. Further attempts to complete the study with microscopic methods could explain the interdependence between organogenesis and cerebellar morphology more clearly.
